# Effect of an Unsupervised Exercises-Based Athletics Injury Prevention Programme on Injury Complaints Leading to Participation Restriction in Athletics: A Cluster-Randomised Controlled Trial

**DOI:** 10.3390/ijerph182111334

**Published:** 2021-10-28

**Authors:** Pascal Edouard, Kathrin Steffen, Marie Peuriere, Pierre Gardet, Laurent Navarro, David Blanco

**Affiliations:** 1 Inter-University Laboratory of Human Movement Science (LIBM EA 7424), University Jean Monnet, University of Lyon, F-42023 Saint Etienne, France; 2 Sports Medicine Unit, Department of Clinical and Exercise Physiology, Faculty of Medicine, University Hospital of Saint-Etienne, 42055 Saint-Etienne, France; 3 Oslo Sports Trauma Research Center, Department of Sports Medicine, Norwegian School of Sports Sciences, 0806 Oslo, Norway; kathrins@nih.no; 4 Délégation à la Recherche Clinique et à l’Innovation (DRCI), Centre Hospitalo-Universitaire (CHU) de Saint-Etienne, 42055 Saint-Etienne, France; Marie.Peuriere@chu-st-etienne.fr; 5 PGC, 42580 L’Etrat, France; pgardet@pg-consultant.com; 6 Mines Saint-Etienne, U1059 Sainbiose, INSERM, University Jean Monnet, University of Lyon, F-42023 Saint-Etienne, France; navarro@emse.fr; 7 Physiotherapy Department, Universitat Internacional de Catalunya, Sant Cugat del Vallès, 08195 Barcelona, Spain; david.blanco@hotmail.com

**Keywords:** sports injury prevention, injury prevention program, athletics, track and field, epidemiology, prospective studies

## Abstract

Objective: To test the efficacy of the Athletics Injury Prevention Programme (AIPP) to reduce the percentage of athletes presenting at least one injury complaint leading to participation restriction (ICPR) over an athletics season. Methods: During the 2017–2018 athletics season, we included in this cluster randomised controlled trial (ClinicalTrials.gov Identifier: NCT03307434) 840 athletes randomly assigned (randomisation unit: athletic clubs) to a control group (regular training) or to an intervention group (regular training plus the AIPP 2/week). Using a weekly online questionnaire, athletes reported the ICPR, training and competition exposures, and, for the intervention group, the compliance with the AIPP. The primary outcome was the percentage of athletes presenting at least one ICPR over the study follow-up. Results: A total of 449 and 391 athletes were included in the intervention and control groups, respectively. From them, 68 (15.1%) and 100 (25.6%) athletes, respectively, provided 100% of the requested information during the follow-up (39 weeks). A total of 6 (8.8%) performed the AIPP 2/week or more. The proportion of athletes who had at least one ICPR over the follow-up period was similar in the intervention (64.7%) and control groups (65.0%), with adjusted odds ratios: 0.81 (95% CI 0.36 to 1.85). There were no between-group differences when comparing separately the subgroups corresponding with the different compliance levels. Conclusion: This cluster randomised controlled trial reported no efficacy of the AIPP. However, the overall response proportion and the compliance with the AIPP in the intervention group were low. In individual sports especially, efforts should be first made to improve the implementation and adoption of interventions.

## 1. Introduction

Athletics (track and field) is an Olympic sport practiced worldwide. Athletics includes several disciplines: sprints, hurdles, jumps, throws, combined events, middle- and long-distance track running, road running (including among others 5 km, 10 km and marathon), and race walking, as well as cross country and mountain and trail running (https://www.worldathletics.org; accessed on 19 October 2021). Participation in athletics invariably leads to a risk of injuries [1]. During any athletic season with a structured prospective injury monitoring, about two third of athletes sustained at least one injury with few variations according to athletes’ age, sex, and disciplines [1,2,3]. The nature of injuries varies according to disciplines, and most frequent reported diagnosis were: hamstring muscle injuries (especially in sprints, hurdles, and jumps), Achilles tendinopathies (especially in sprints, jumps, middle- and long-distances), knee overuse injuries including patellar tendinopathies (especially in sprints, middle- and long-distances), shin splints and stress fractures (especially in sprints, middle- and long-distances), ankle sprains (especially in jumps and throws), and lower back pain (especially in jumps and throws) [1,2,3,4,5,6]. In many ways, injuries in athletics, directly or indirectly, affect an athletes’ training, performance, career, and/or health negatively [7,8,9]. The reduction in injury risk is thus fundamental to promote healthy and sustainable athletics practice.

The lack of studies analysing injury risk reduction programmes in athletics motivated Edouard et al. [10] to develop and analyse an exercises-based injury risk reduction programme. The Athletics Injury Prevention Programme (AIPP) was developed based on the literature on the epidemiology of athletics injuries, injury risks factors, and current evidence-based exercises-based injury prevention programmes [10], and aimed to target the most common athletics injuries (hamstring muscle injuries, Achilles and patellar tendinopathies, low back pain, ankle sprains) [1,2,3]. The AIPP included strengthening, stretching and neuromuscular control exercises aiming at targeting the most common athletics injuries [10]. The AIPP revealed promising findings in being significantly associated with lower risk of injury complaints related to athletics practice that leads to restrictions in athletics participation (called then “injury complaint leading to participation restriction” (ICPR)) in the short (12 weeks), but not in the long (40 weeks) terms [10]. Although this was the first prospective cohort study (level of evidence 2) on injury risk reduction programme designed specifically for athletics, the study included a small sample size (62 athletes) and needed to be replicated through a randomised controlled design (RCT).

In this context, the primary aim of this cluster RCT was to test the efficacy of the Athletics Injury Prevention Programme (AIPP) to reduce the percentage of athletes presenting at least one ICPR over an athletics season. The secondary aims of the study were to test the efficacy of the AIPP to reduce ICPR burden (i.e., number of days lost per 1000 h of exposure [11]) and whether the AIPP increased time (weeks) before athletes become injured for the first time during an athletics season.

## 2. Methods

### 2.1. Study Design and Overall Procedure

From October 2017 to July 2018 (40 weeks of athletics season), we conducted a researcher-blinded unsupervised cluster RCT (called “PREVATHLE”) including competitive athletes who were randomly assigned (allocation ratio 1:1) to a control group where they continued their regular training or to an intervention group where we added the AIPP to their regular training. The study protocol was reviewed and approved by the Committee for the Protection of Persons (CPP Ouest II—Angers, number: 2017-A01980-53) and by the French Federation of Athletics (FFA, http://www.athle.fr; accessed on 19 October 2021) prior to recruitment, and was registered on ClinicalTrials.gov (Identifier: NCT03307434). To produce the present manuscript, we used the Consolidated Standards of Reporting Trials (CONSORT) [12]. Athletes and the public were not involved in the trial design and conduct of the study or the choice of outcome measures.

### 2.2. Population Recruitment and Inclusion/Exclusion Criteria

At the start of the 2017–2018 athletics season, the FFA sent the 23 October 2017 through email to all athletes licensed at the FFA for competition an invitation to participate in this RCT at an individual level. Detailed information about the study purpose and procedure, participant rights and contact information for further questioning was available in the email. Athletes were invited to register online using a specific secured website called “Prevathle” (Windows Server 2013 R2 64 bits—SP2; IBM DOMINO 9.01 fix pack 8) to proceed with the inclusion.

The inclusion criterion of a cluster was a club with at least 15 licensed athletes. As the extra materials are usually present in most athletic clubs and were only required for the higher levels of the AIPP, we did not ask the clubs whether they actually had these materials, and this did not represent a cluster inclusion criterion.

The inclusion of athletes in the study was performed electronically, during a two-week period (from 23 October 2017 to 6 November 2017), based on the following criteria: athletes must be licensed at the FFA in a club of at least 15 athletes (i.e., included cluster), without any contraindications for competitive athletics activity attested by the license at the FFA, aged between 15 to 40 years, and having access to the Internet. We excluded athletes if they declined to participate in the study or if they were unable to express agreement or signing the informed consent. We did not exclude athletes based on their baseline injury status or history [10].

If they met the inclusion criteria, athletes had to provide written informed consent for participation, as well as their parents for those under 18 years old.

### 2.3. Randomisation

After the inclusion, we randomised each athlete to the intervention or the control group at a club level to minimise the risk of contamination bias between athletes from the same club. An independent statistician performed this cluster randomisation a priori using SAS 9.4 (SAS Institute, Inc., Cary, NC, USA) in a 1:1 ratio that assigned a unique randomisation number to each included athlete. The randomisation was stratified according to the number of athletes and the national ranking of each club.

### 2.4. Interventions

Apart from their regular training, we asked athletes in the intervention group to perform the Athletics Injury Prevention Programme (AIPP) at least twice a week [10]. The AIPP included 8 exercises with levels of progression (from 2 to 5 depending on the exercise): core stability (plank and side plank), postural control (one-leg balance), pelvic strengthening (lunges and hip abductor strengthening), hamstring exercises (stretching and isometric, concentric and eccentric strengthening), and lower leg exercises (stretching and eccentric strengthening) [10]. The specific exercises, number of repetitions, and levels of progression are presented in Table 1, and detailed information on the AIPP is provided in (supplementary file Figure S1). Only for athletes in the intervention group, we sent the AIPP via e-mail in both paper (Figure S1) and video versions. The AIPP was also available on the website used for data collection. No further guidance was given. Once the athletes were familiar with the exercises, the AIPP took about 15 min to complete.

We asked athletes in the control group to follow their regular training plan.

### 2.5. Data Collection

At the start of the season, we collected baseline information on each included athlete using a survey developed in Google Forms (Google^®^): sex, age, height, body mass, discipline, usual week hours of athletics practice, and history of ICPR during the preceding season (yes or no).

During the season, we automatically sent an e-mail every Monday with a secured link to the weekly online questionnaire to all included athletes. It aimed to collect information on the preceding week: number of hours of training and competition (i.e., athletics exposure), number of complete AIPP sessions performed, and possible injury complaints. We sent two automatic reminders 3 and 5 days after the first e-mail to non-responders. The data were collected using a secured website called “Prevathle” (Windows Server 2013 R2 64 bits—SP2; IBM DOMINO 9.01 fix pack 8). We calculated the response proportions by dividing the number of completed weekly questionnaires by the maximum number of questionnaires expected to be completed.

### 2.6. Injury Definition

We chose the term “injury complaint”, as mentioned in the study from Edouard et al. [10], since it refers to self-reported information without medical diagnosis [13], usually defined as: “a pain, physical complaint or musculoskeletal lesion sustained by an athlete during participation in athletics training or competition, regardless of whether it received medical attention or its consequences with respect to impairments in connection with competition or training” [14].

Athletes reporting an injury complaint were asked to provide the following information: circumstance of injury occurrence (training (including during the AIPP), competition, outside of athletics), mode of onset (sudden or gradual) [14], injury location [14], and its consequence on athletics participation with four categories [15,16]: full participation with no discomfort, full participation with discomfort, reduced participation due to injury complaint, full absence from sport due to injury complaint). In this study, the term “injury complaint leading to participation restriction” (ICPR) corresponds to the last two categories (reduced participation or full absence in athletics).

### 2.7. Compliance

We asked athletes to report an AIPP session only if they had performed all the 8 exercises of the programme. For each athlete, we measured compliance with the AIPP as the average number of AIPP sessions performed per week. We categorized compliance as follows: good (at least 2 weekly AIPP sessions, as requested), moderate (between at least 1 to less than 2 weekly AIPP sessions), and low (less than 1 weekly AIPP session). We monitored adverse events of the AIPP, and especially ICPR, through the weekly online questionnaire.

### 2.8. Blinding

The independent statistician who performed the cluster randomisation was blinded to the study protocol. The assessment and the delivery of the intervention were blinded as all data were collected via an online questionnaire, and the intervention was delivered online through e-mail and the website, ensuring no influence or bias of healthcare providers or assessors of outcomes. The principal investigator (PE) and the statistician (DB) were also blinded, and outcome measures were not available to any party until all data had been collected. However, it was not possible to blind athletes and coaches.

### 2.9. Sample Size

We used MedCalc (https://www.medcalc.org; accessed on 23 May 2017) for the sample size calculation. We expected to reduce the average percentage of athletes injured at least once per season from ~70%, as described in the literature [1,2], to 50% using the AIPP. Given a power of 90%, a significance level of 5%, an expected cluster size of 15 athletes per club with an inflation factor of 1.7 (calculated as 1 + ICC*(m-1), where ICC = 0.05 and m = 15), and a dropout rate of 40%, we calculated that we needed to recruit a total of 704 athletes (352 per group).

### 2.10. Study Outcomes

The primary outcome was the percentage of athletes who presented at least one ICPR during the 40 weeks of the athletics season. The secondary outcomes are the ICPR burden over an athletics season, defined as the number of days lost due to ICPR per 1000 h of exposure [11], and the time (in weeks) between the first week of the study and the week of the first ICPR. All these variables were calculated using data from the weekly online questionnaire.

### 2.11. Statistical Methods

We carried out statistical analysis using R V.3.6.0 (version 3.6.0, ©Copyright 2016 The Foundation for statistical Computing (Comprehensive R Archive Network, http://www.R-project.org; accessed on 2 July 2021). Significance was accepted at *p* < 0.05.

For the primary outcome, we adjusted a multivariate logistic regression model where the dependent variable was the occurrence (or non-occurrence) of at least one ICPR, and the independent variables were the participant’s allocation, compliance with the AIPP, athletics exposure, club, sex, age, and history of ICPR during the previous season. Following the purposeful selection approach described by Hosmer and Lemeshow [17], which includes performing univariate analyses for each of the dependent variables, we included in the final model those covariates of special clinical relevance (participant’s allocation and compliance), as well as the ones that result in a better model [17]. In this analysis, we only included athletes with 100% of weekly response proportion.

For the secondary outcomes, we first adjusted a linear regression model where the dependent variable was ICPR burden and followed the same process described above, only considering athletes with 100% response proportion.

Finally, we used a survival analysis to evaluate the time to the first ICPR. For athletes who reported no ICPR during athletics practice, we censored them (1) at the time of their last questionnaire submission in case they stopped completing the weekly questionnaire, (2) when they had a ICPR that occurred outside of athletics practice, or (3) at the end of the study (40 weeks) if they submitted all questionnaires and had no ICPR outside of athletics. First, we carried out a competing risk analysis (using R package “cmprsk”) to determine whether there were significant differences between the control and intervention groups in terms of the cumulative incidence of ICPR occurred during athletics and outside of athletics [18]. Second, we adjusted a Cox proportional hazards model and followed the variable selection process described above.

For these three analyses, we also performed subgroup analyses for all variables of interest based on the degree of compliance with the AIPP (good, moderate, or low).

### 2.12. Deviations from the Protocol

We did not conduct all the secondary analyses planned in the Clinicaltrials.gov (gov Identifier: NCT03307434; accessed on 19 October 2021). In particular, no analyses at 12 weeks of follow-up have been performed. Due to an informatic problem which did not allow the last weekly automatic email to send, the entire period of follow-up was 39 weeks instead of the planned 40 weeks. We included more athletes than calculated a priori to allow us enough power for subgroup analyses.

## 3. Results

### 3.1. Population

**Figure 1:**
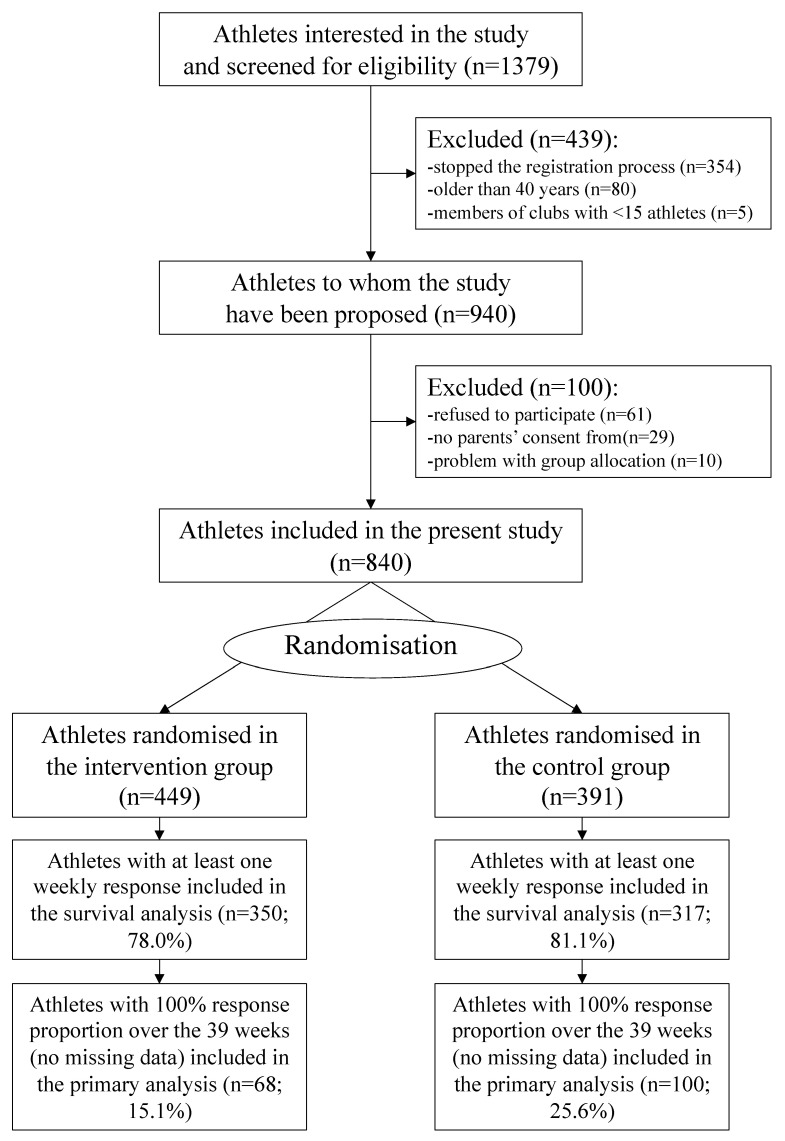
The CONSORT flow diagram of the study.

Among all invited athletes, 1379 athletes went on to the website, and 449 athletes allocated to the intervention group and 391 to the control group were finally included (baseline characteristics in Table 2). The intervention and control groups were balanced in terms of the main clinically relevant variables, for the total population and the two subgroups used for analyses, and no baseline hypothesis testing was undertaken as suggested by the CONSORT group [12]. Figure 1 shows the study flow diagram.

### 3.2. Response Proportions to the Weekly Questionnaire

**Figure 2:**
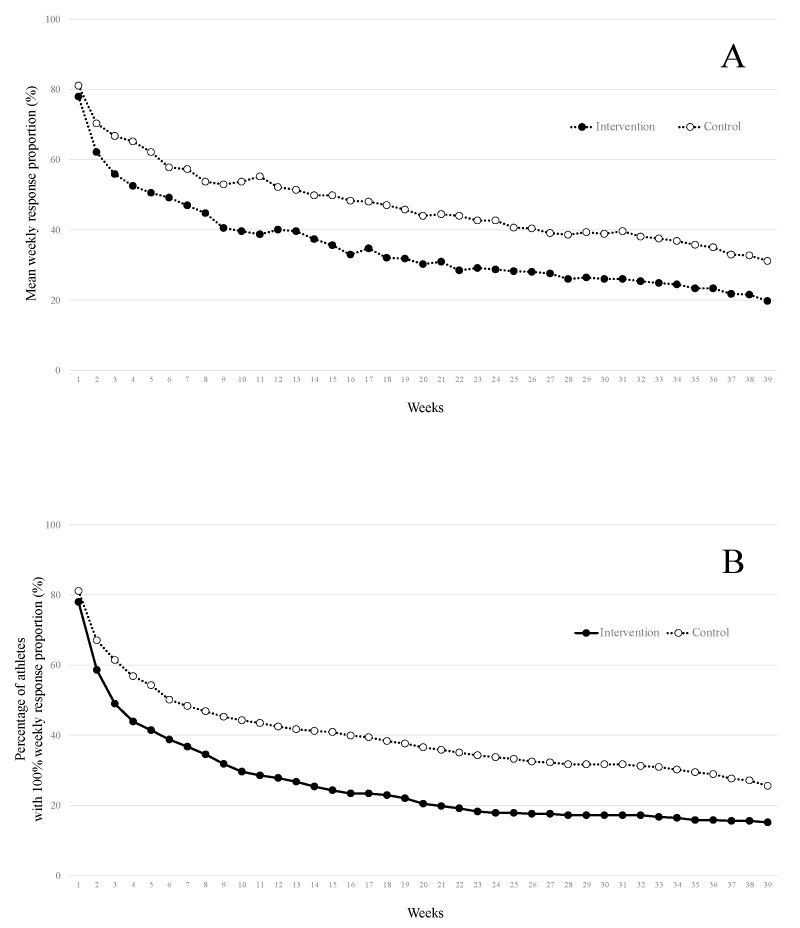
The evolution of (**A**) the mean weekly response proportions and (**B**) the percentage of athletes with 100% of response proportions in the intervention and the control groups over the 39 weeks of follow-up.

Over the 39 weeks, 379 (84.4%) in the intervention and 340 (87.0%) in the control groups provided at least one response. Among the 840 included athletes, 99 (22.0%) in the intervention group and 74 (18.9%) in the control group did not provide any response for the first week of follow-up. The average individual weekly response proportions over the 39 weeks were (mean (SD)) 35.0 (38.8%) in the intervention group and 47.3 (42.4%) in the control group, range from 0.0 to 100.0% in both groups. A total of 168 athletes provided 100% of the responses: 68 (15.1%) in the intervention and 100 (25.6%) in the control groups. The mean weekly response proportions, as well as the number of athletes with 100% of weekly response proportion, continuously decreased during the follow-up (Figure 2).

### 3.3. Compliance with the AIPP and Adverse Effects

Among the 68 athletes in the intervention group with 100% of weekly response proportion, 6 (8.8%) had good compliance respecting the study recommendation, 26 (38.2%) had moderate compliance, and 36 (52.9%) had low compliance (Table 3 and Table 4).

Among the 350 athletes in the intervention group included in the survival analysis, 43 (12.3%) had good compliance respecting the study recommendation, 76 had moderate compliance (21.7%), and 231 had good compliance (66.0%) (Table 5).

No ICPR nor adverse events were reported to occurred during the AIPP; only one athlete reported aggravation of an adductor tendinopathy following lunges.

### 3.4. Primary Outcome Results

The proportion of athletes who had at least one ICPR over the 39-week follow-up period was similar in the intervention (64.7%) and control groups (65%), adjusted odds ratio (OR): 0.81 (95% CI 0.36 to 1.85) (Table 3). There were no between-group differences when analysing separately the subgroups according to compliance with the AIPP (Table 3).

### 3.5. Secondary Outcome Results

We observed no differences in ICPR burden between the intervention (mean 343.3 days lost per 1000 of exposure, SD 817.8) and the control groups (mean 285.6 days, SD 619.6), an adjusted difference of 11.3 days (95% CI −254.3 to 276.9) favouring the control group, and no differences in the compliance subgroup analyses (Table 4).

**Figure 3:**
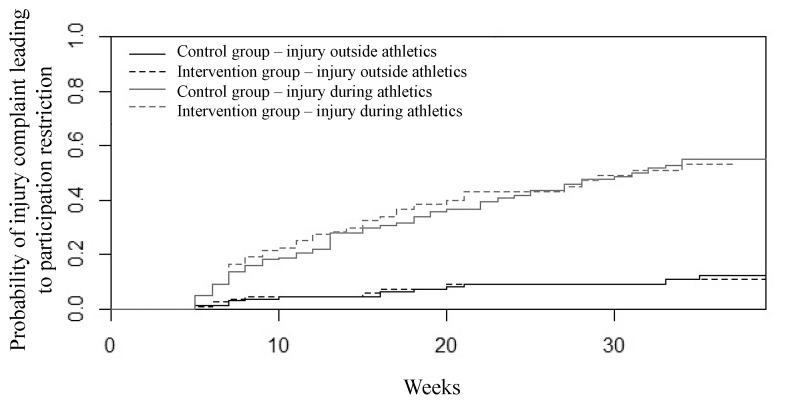
Cumulative incidence curves for the intervention (dotted line) and control (full line) groups for the probability of ICPR occurred during athletics (in grey) and outside of athletics (in black).

Regarding the survival analysis, the competing risk analysis showed that cumulative incidence curves for the two study groups are not statistically different for ICPR outside of athletics (*p* = 0.11), nor for ICPR during athletics (*p* = 0.70) (Figure 3). When we performed this comparison between levels of compliance with the AIPP (good adherence versus moderate and low adherence together, and good and moderate together versus low), the cumulative incidence curves for the two groups were not significant for each of the two types of ICPR.

The survival analysis yielded no differences in the time to the first ICPR between the intervention (mean 6.62 weeks, SD 9.64) and the control groups (mean 7.58 weeks, SD 8.94), and adjusted HR 1.02 (95% CI 0.76 to 1.37), with similar results in the compliance subgroup analysis (Table 5).

## 4. Discussion

The main finding of the present study was that there were no significant differences between the intervention group and the control group for (i) the percentage of athletes presenting at least one ICPR, (ii) the ICPR burden, and (iii) the time before athletes become injured for the first time, over an athletics season. It is also important to note that the overall response proportion and the compliance with the AIPP in the intervention group were low, which could have, among other reasons, influenced the results. These issues represent an important challenge to be considered in future studies.

### 4.1. Challenge of Low Response Proportion to the Weekly Questionnaire

The mean response proportion was less than 50%, which was inferior to that reported in previous epidemiological studies in elite athletes (69–91%) [2,19,20], but closer to that in lower level athletes (50–60%) [21,22]. Possible explanations could be the athletes’ level (all levels vs. national and international levels in other studies [2,19]), the culture (*Latin* in our and Barboza et al. [21] studies vs. *Nordic* [2,19,20]), the few feedbacks to athletes [21], or the specificities of athletics as individual sports [2,23]. As previously reported [15,21,22], we observed a decrease in the response proportions throughout the follow-up period.

The low response proportions of athletes to regular self-reported questionnaires and its decrease with time represent major challenges in epidemiological and interventional studies, especially in athletics [23], as these studies are dependent on high data quality. Efforts should be made to reach a high level of response proportion. In addition to regular automatic reminders to athletes, other strategies should be developed, for instance, having one investigator monitoring the response proportion during the study and trying to enforce participation, educating end-users on the interest of monitoring the evolution of some characteristics of athletes over the time (e.g., training load, pain, fatigue), providing some visual feedbacks of these characteristics and evolutions, and providing scientific evidence of the improvement of health and/or performance by using this monitoring [19,21].

### 4.2. Challenge of Compliance with the Intervention

The first lesson that we learnt was the low compliance of athletes with the unsupervised exercises-based injury prevention programmes: only 8.8% were fully compliant. This was consistent with those from previously published RCT [24,25], and a recent RCT analysing an unsupervised tennis-specific prevention programme (8% of participants with high compliance) [22]. This issue can be explained by the nature of individual sports, which makes implementing an intervention and conducting RCTs difficult [22,24,26] in comparison with team sports [20,27].

In this context of low compliance, it is recommended to use appropriate statistical approaches, such as instrumental variable analysis [24,28]. However, as previously discussed [28], instrumental variable analysis cannot be applied to the present dataset. Therefore, we followed a more standard approach, including analyses of compliance sub-groups.

Future studies should take into account the potential low compliance with the intervention proposed. This could in part be explained by its unsupervised nature [22]. Therefore, face-to-face or online supervision of the programme could be a way to improve compliance. Increasing athletes’ motivation to carry out the programme through incentives could also be another way to go. Incentives could include money, discounts for sporting equipment, free access to competition as a participant or spectator, or free books or online information on athletics or athletics injury prevention. Additionally, providing athletes with further education in addition to the paper and video AIPP documents could help with improving compliance. Education could be (i) specific to the AIPP, for instance, explaining why it was created, why such exercises are included in the programme, or what is expected by conducting it, and (ii) more general on the interest of health protection and injury prevention in sports. In general, more efforts could be invested on the implementation, adoption, and adherence to the programme [29,30], particularly in individual sports [22,24,26]. This can be carried out, for instance, by adding the structured step-by-step approach described by Owoeye et al. [31] to the development of the intervention, including the identification of compliance rate, determination of compliance/non-compliance predictors, and developing strategies to improve compliance, which could be tested in pilot studies.

### 4.3. The Athletics Injury Prevention Programme

The present study does not report efficacy of the AIPP to reduce the injury risk, in line with other unsupervised prevention programme [22,26]. Except one prospective cohort study analysing the AIPP in athletics [10], there is currently and to our knowledge no other studies in athletics (track and field) analysing exercise-based injury prevention programmes that could be compared with our present study. In addition, given the low compliance and the reported lack of efficacy of the programme, we chose not to compare our present results with those from RCT in other sports.

Apart from the low response proportion and compliance issues discussed above, which can disguise the real effect of the AIPP, it is also possible that the AIPP itself may be insufficient to reduce the injury risk. While its content has been developed based on the literature on the epidemiology of athletics injuries, injury risks factors, and current evidence-based exercise-based injury prevention programmes [10], and targets the frequency and volume that have been reported to be efficient to reduce injury risk (at least two times a week for about 15 min) [32], the current programme did not achieve a reduction in injury risk. The fact that some athletes in both groups could already be performing the proposed exercises as part of their training routine might have attenuated the effect of the intervention. In the long term, as reported by Edouard et al. [10], the current programme could not be appropriate enough to improve neuromuscular capabilities that play a role in reducing injury risk. Efforts could be made to improve the content of the programme. For instance, the difficulty of the programme should increase throughout the season. Additionally, it should be tailored to each discipline, and it should target the individual athlete’s characteristics and deficiencies [10]. Athletes, coaches, and other stakeholders could be involved in the programme development [22,30].

### 4.4. Strengths and Limitations

A major strength of this study is the RCT design (level of evidence 1) and one of the first on injury risk reduction programme in individual sports setting [22,26], and the first in athletics (except running). Another strength is the method used for data collection [10]. We also chose to perform the primary analysis on athletes with 100% of data, since missing data can lead to bias, and data imputation of the outcome (i.e., ICPR) means that we are able to predict the outcome, which is currently and to our knowledge not possible. We clearly defined and evaluated the compliance [24,25]. Finally, in a practical point of view, the AIPP is inexpensive to implement, easy to include in training schedules, easy to perform, and unlikely to harm the athlete.

The main limitations of the present study were (i) the low response proportion to the weekly questionnaire leading a smaller number than expected of athletes included in the primary analysis, which could have led to a higher risk of type II error, and (ii) the small percentage of athletes from the intervention group compliant with the prevention programme making that we did not formally compare a group of athletes carrying out the programme vs. not. Another limitation was our lack of quality control when athletes performed the AIPP. Finally, the self-reported data collection also limited the amount of information provided regarding injury diagnoses [27].

### 4.5. Practical Implications

Given our findings, it is not possible to conclude the efficacy of the AIPP to reduce the risk of injuries in athletics, and consequently to promote its use. However, the AIPP does not appear to present adverse effects, meaning injuries/discomfort by carrying out the AIPP. The present findings do not allow us to disseminate the AIPP as a useful efficient injury risk reduction programme, but it can maybe be used as a basis for the development of future programmes. Future programmes should target the most common athletics injuries and their modifiable risk factors and mechanisms, be specific to the discipline and to the individual athlete’s characteristics, be easily integrated in usual training and/or daily life, and provide benefits on sports practice and performance.

Another important lesson learnt is the need to improve the implementation, delivery, receipt, and adoption of any injury risk reduction programme, especially in individual sports [22,26] such as athletics. This could be performed by (1) educating athletes and all stakeholders around them on the interest of reducing the injury risk, (2) asking athletes, coaches and other stakeholders to actively collaborate or lead the development of injury risk reduction programmes [22,29,30], (3) focusing the efforts on developing programmes that could be inexpensive to implement, easy to include and perform in training schedules or daily life, and with potential benefit for performance, and (4) improving the implementation/dissemination of the programme based on behaviour change determinants and principles [33].

## 5. Conclusions

The present cluster RCT reported no significant differences between the intervention group and the control group for (i) the percentage of athletes presenting at least one ICPR, (ii) the ICPR burden, and (iii) the time before athletes become injured for the first time, over an athletics season. It is also important to note that the overall response proportion and the compliance with the AIPP were low. Perspectives are the improvement of the AIPP content and its implementation strategy.

## Figures and Tables

**Table 1:** Content and structure of the Athletics Injury Prevention Programme.

Exercises	Repetitions	Bonus (If It Is Too Easy, Athlete Can Increase Difficulty)
**Core stability—the plank (4 sides: prone position, lateral, supine position, lateral):**		
Level 1: both legs	15 sec. per side for 3 min	4 × 30 sec. for 6 min, then for 12 min
Level 2: alternate legs		
Level 3: unstable support		
**Single leg balance:**		
Level 1: static	3 × 15 sec. (each side)	3 × 30 sec. each side
Level 2: unstable support		
Level 3: throwing ball with partner, then on unstable support		
**Pelvis strengthening:**		
Lunges	3 × 10 rep.	6 × 10 rep., then 6 × 10 rep. with medicine ball
Hip abductor strengthening—Level 1: leg empty	3 × 10 rep. (each side)	6 × 10 rep. (each side), then unstable support
Hip abductor strengthening—Level 2: with elastic	3 × 10 rep. (each side)	6 × 10 rep. (each side), then unstable support
**Hamstring exercises:**		
Hamstring stretching (different positions)	3 × 15 sec.	
Hamstring strengthening		
Level 1: isometric contraction on two legs	6 × 6 sec.	10 × 10 sec.
Level 1: heel to buttock with elastic	3 × 6 sec. (each side)	5 × 10 sec. (each side)
Level 2: isometric contraction on one leg	6 × 6 sec. (each side)	10 × 10 sec. (each side)
Level 2: heel to buttock with elastic	3 × 6 sec. (each side)	5 × 10 sec. (each side)
Level 3: Nordic hamstring with help of upper arms	1 × 5 rep., then 3 × 5 rep., then 6 × 6 rep.	
Level 4: Nordic hamstring	1 × 5 rep., then 3 × 5 rep., then 6 × 6 rep.	
Level 5: Pliometric	1 × 5 rep., then 3 × 5 rep., then 6 × 6 rep.	
**Lower leg exercises:**		
Lower leg stretching	3 × 15 sec. (each side)	
Lower leg strengthening—Level 1: down to the ground	3 × 8 rep. (each side)	3 × 10 rep., then 5 × 10 rep. each side and then increase load
Lower leg strengthening—Level 2: down to the void	3 × 8 rep. (each side)	3 × 10 rep., then 5 × 10 rep. each side, and then increase loads

sec., seconds; rep., repetitions.

**Table 2:** Baseline characteristics at the individual level of the athletes included. Data are presented as mean and standard deviation (SD) for continuous variables, and numbers and percentages (%) for ordinal or categorical variables.

	Total Included Athletes (*n* = 840)				Athletes with 100% Response Proportion over the 39 Weeks (*n* = 168)				Athletes Included in the Survival Analysis with at Least One Weekly Response (*n* = 667)			
	Intervention (*n* = 449)		Control (*n* = 391)		Intervention (*n* = 68)		Control (*n* = 100)		Intervention (*n* = 350)		Control (*n* = 317)	
Sex:												
Female athletes	201	(44.8)	149	(38.1)	28	(41.2)	29	(29.0)	155	(44.3)	115	(36.3)
Male athletes	248	(55.2)	242	(61.9)	40	(58.8)	71	(71.0)	195	(55.7)	202	(63.7)
Age (years)	30.1	(6.7)	30.2	(6.6)	32.4	(6.1)	30.9	(6.5)	30.4	(6.7)	30.5	(6.4)
Height (cm) *	172.5	(8.7)	173.0	(8.1)	173.5	(8.6)	175.3	(8.2)	172.5	(8.9)	173.3	(8.1)
Body mass (kg) *	63.2	(10.1)	64.3	(11.6)	63.2	(9.3)	64.3	(9.2)	63.1	(10.3)	64.7	(11.6)
Disciplines: *												
Endurance	347	(77.3)	305	(78.0)	60	(88.2)	79	(79.0)	279	(79.7)	248	(78.2)
Road running	185	(41.2)	162	(41.4)	30	(44.1)	38	(38.0)	144	(41.1)	130	(41.0)
Middle and long distances running (track)	83	(18.5)	60	(15.3)	15	(22.1)	19	(19.0)	71	(20.3)	49	(15.5)
Race Walking	2	(0.4)	7	(1.8)	1	(1.5)	2	(2.0)	2	(0.6)	5	(1.6)
Trail	77	(17.1)	76	(19.4)	14	(20.6)	20	(20.0)	62	(17.7)	64	(20.2)
Explosive	97	(21.6)	84	(21.5)	8	(11.8)	21	(21.0)	70	(20.0)	67	(21.1)
Sprints	44	(9.8)	30	(7.7)	4	(5.9)	4	(4.0)	37	(10.6)	22	(6.9)
Hurdles	13	(2.9)	12	(3.1)	1	(1.5)	4	(4.0)	9	(2.6))	9	(2.8)
Jumps	19	(4.2)	16	(4.1)	2	(2.9)	4	(4.0)	13	(3.7)	14	(4.4)
Throws	14	(3.1)	14	(3.6)	0	(0.0)	2	(2.0)	8	(2.3)	10	(3.2)
Combined events	7	(1.6)	12	(3.1)	1	(1.5)	7	(7.0)	3	(0.9)	12	(3.8)
Weekly athletics training (hours) *	5.4	(2.5)	5.7	(2.9)	5.3	(2.1)	5.3	(2.4)	5.3	(2.4)	5.6	(2.7)
Week sport practice expect athletics (hours) *	2.8	(3.0)	2.5	(2.7)	2.5	(2.9)	2.2	(2.5)	2.7	(2.9)	2.4	(2.6)
History of ICPR during the preceding season: *												
No	140	(52.0)	142	(54.4)	36	(52.9)	53	(53.0)	124	(52.5)	132	(54.3)
Yes	129	(48.0)	119	(45.6)	32	(47.1)	47	(47.0)	112	(47.5)	111	(45.7)

ICPR: injury complaint leading to participation restriction. * Note that baseline information is missing for some variables: For the total of 840 included athletes, information was missing for height and body mass (178 athletes in the intervention and 127 in the control groups), for disciplines (5 intervention and 2 control), for weekly athletics training practice and sport practice except athletics and history of injury complaints during the preceding season (180 intervention and 130 control). For ICPR during the preceding season, the percentage are calculated based on the total number of responders (269 (59.9%) intervention and 261 (66.8%) control). For the 667 included in the survival analysis, information was missing for disciplines (1 intervention and 2 control), for height, body mass, weekly athletics training practice and sport practice expect athletics, and history of participation restriction injury complaints during the preceding season (114 intervention and 74 control). For ICPR during the preceding season, the percentages are calculated based on the total of responders (236 (67.4%) intervention and 243 (76.7%) control).

**Table 3:** Primary outcome results: comparison of the proportion of athletes who had at least one IPCR over the follow-up period (39 weeks) between the intervention and the control groups using an adjusted logistic regression model where the dependent variable was the occurrence (or non-occurrence) of at least one ICPR, and the dependent variables were the participant’s allocation, compliance with the AIPP, athletics exposure (training and competition), club, sex, age, and history of ICPR during the previous season.

Outcome		Percentage (No)		Adjusted Odds Ratio (95% CI) *
		Intervention Group (*n* = 68)	Control Group (*n* = 100)	
Proportion of athletes who presented at least one ICPR over 39 weeks (*n* = 168)		64.7% (*n* = 44)	65.0% (*n* = 65)	0.81 (0.36 to 1.85)
	Low compliance ** (*n* = 36 of 68)	63.9% (*n* = 23 of 36)		0.85 (0.37 to 1.97)
	Moderate compliance ** (*n* = 26 of 68)	69.2% (*n* = 18 of 26)		1.13 (0.45 to 3.04)
	Good compliance ** (*n* = 6 of 68)	50.0% (*n* = 3 of 6)		0.75 (0.12 to 4.82)

* OR < 1 favours intervention, >1 favours control. OR is adjusted by AIPP compliance, athletics exposure (training and competition), and occurrence (or non-occurrence) of at least one injury in the previous season. ** Only considers the intervention group. ICPR: injury complaint leading to participation restriction; SD: standard deviation; CI: confidence interval.

**Table 4:** Secondary outcome results: comparison of the number of days lost due to injury complaint leading to participation restriction (ICPR) per 1000 h of athletics exposure.

Outcome		Intervention Group (*n* = 68) (Mean (SD)	Control Group (*n* = 100) (Mean (SD)	Adjusted Difference * (95% CI)
Injury burden (days lost per 1000 h of exposure) over 39 weeks (*n* = 168)		343.3 (817.8)	285.6 (619.6)	11.3 (−322.5 to 172.9
	Low compliance ** (*n* = 36 of 68)	307.1 (513.2)		19.9 (−207.5 to 247.3)
	Moderate compliance ** (*n* = 26 of 68)	449.6 (1180.2)		134.2 (−200.3 to 468.8)
	Good compliance ** (*n* = 6 of 68)	99.9 (125.3)		−159.2 (−670.2 to 351.8)

* Positive differences favour control; negative differences favour intervention. Differences are adjusted by AIPP compliance, and occurrence (or non-occurrence) of at least one injury in the previous season. ** Only considers the intervention group. ICPR: injury complaint leading to participation restriction; SD: standard deviation; CI: confidence interval.

**Table 5:** Secondary outcome results: survival analysis to evaluate the time before the first participation restriction injury complaint leading to participation restriction (ICPR) (in weeks).

Outcome		Intervention Group (*n* = 350) (Mean (SD)	Control Group (*n* = 317) (Mean (SD)	Adjusted Hazard Ratio * (95% CI)
Time to the first ICPR during athletics practice (in weeks) (*n* = 667)		6.62 (9.64)	7.58 (8.94)	1.02 (0.76 to 1.37)
	Low compliance ** (*n* = 231 of 350)	5.62 (8.94)		0.96 (0.65 to 1.40)
	Moderate compliance ** (*n* = 76 of 350)	9.34 (11.41)		1.06 (0.60 to 1.89)
	Good compliance ** (*n* = 43 of 350)	7.19 (9.11)		0.90 (0.43 to 1.90)

* OR < 1 favours intervention, >1 favours control. OR is adjusted by AIPP compliance, athletics exposure (training and competition), and occurrence (or non-occurrence) of at least one injury in the previous season. ** Only considers the intervention group. ICPR: injury complaint leading to participation restriction; SD: standard deviation; CI: confidence interval.

## Data Availability

Data are available upon reasonable request. Requests for data sharing from appropriate researchers and entities will be considered on a case-by-case basis. Interested parties should contact the corresponding authors Pascal Edouard (pascal.edouard@univ-st-etienne.fr).

## Supplementary Materials

The following are available online at https://www.mdpi.com/article/10.3390/ijerph182111334/s1, Figure S1: The Athletic Injury Prevention Programme: a detailed programme for athletes and coaches.
