# Supplementary material for: Effect of an Unsupervised Exercises-Based Athletics Injury Prevention Programme on Injury Complaints Leading to Participation Restriction in Athletics: A Cluster-Randomised Controlled Trial
Source: Int J Environ Res Public Health. 2021 Oct 28;18(21):11334. doi: 10.3390/ijerph182111334 (PMC8583284; doi:10.3390/ijerph182111334)
Supplement: Supplementary file 1 [file ijerph-18-11334-s001.zip › ijerph-1414182-supplementary.pdf]

Athletics Injury Prevention Program (AIPP)

The programme of exercises is similar for all athletes

When? *The exercises are to be performed during or at the end of the warm-up, but can also be done at the end of the training session, or as an exercise session in its own right following a bike/run warm up.*
Which frequency? *2 times a week in a training-only period, and once a week in competition period.*
How many time? *AIPP takes about 15 minutes.*
How to move to the next step? *When you are able to perform an exercise without difficulty, you can move on to the higher difficulty level (count about 1 month per level).*
What to do if it is too easy? *You can increase the duration and / or repetition of the exercise (part: "BONUS").*

Of course, all other muscle strengthening, balance and stretching exercises are encouraged.

1. Core stability ("the plank on 4 sides")

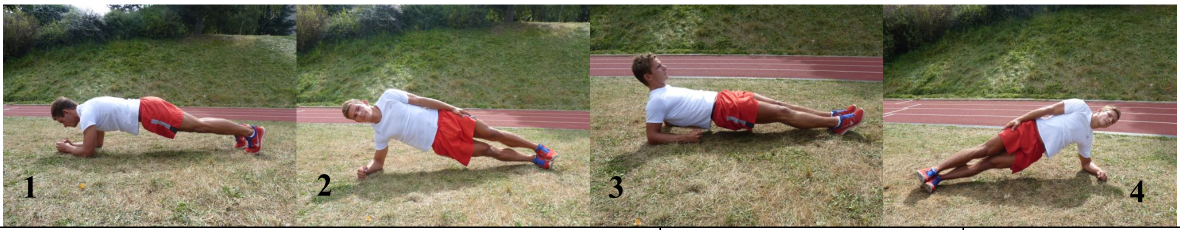

Level 1:	Level 2:	Level 3:
Prone position (1): Forearm plank, forearms parallel to each other, elbows should be beneath shoulders, vertically. Lift the body, resting on the forearms, tuck in the belly, to form a straight line with the body. Hold the pose while breathing smoothly. Side forearm plank (2 and 4): keep on elbows and feet, the body must be straight and form a triangle with the field. Reverse forearm plank (3): keep on elbows, on a supine position, the body straight. Lock the abdominals, tighten gluteus muscles, keep your back flat and try not to wobble.	Same position with only one support for the upper and lower limbs for the prone position (raising an arm and the opposite leg), and for the lateral side position with hip and knee at 90 °.	Same position with support of the upper or lower limbs on a ball or an unstable support.
15 seconds per side for 3 minutes.		
BONUS: increase to 4x30 seconds for 6 minutes, then for 12 minutes		

2. Single leg balance

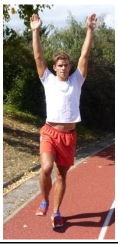

Level 1:	Level 2:	Level 3:
Static: one foot on the ground	Dynamic: one foot on an unstable support.	Double task: Throwing ball with a partner when on one foot, then on an unstable support.
3x15 seconds per side		
Note: For each level, vary positions at each session: knee bent or straightened, hands on hips or arms stretched vertically, eyes open or eyes closed (except for passes only with open eyes), on tiptoe ...		
BONUS: increase to 3x30 seconds per side		

3. Pelvic strengthening (Hip adductors and abductors)

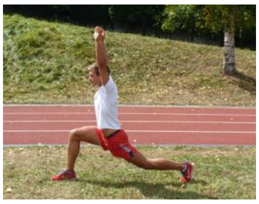

Lunges (Adductors): From a standing position, slowly perform a lunge by tacking a big stride forward. When performing the lunge, advance the front leg as far as possible until the hip and knee are bent at 90 degrees and the back leg is as far back as possible. Recline only on the front leg. Keep the knee in line, back straight and look front. Try to keep the upper body and hips stable. The hands may be on the hips or arms stretched vertically.

3x10 lunges. *(Note: for beginners 3x5 lunges)*

BONUS: 6x10 lunges, then 6x10 lunges medicine ball.

Level 1:

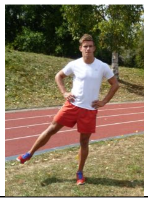

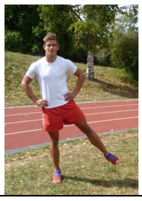

Abductors: When resting on one foot, an abduction of the hip is performed and the position is held for 2 seconds and gently released. Initially, the upper limbs can help for balance.

Level 2:

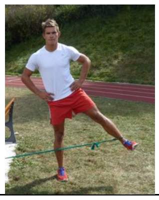

Same with a rubber band.

3x10 repetitions each side

BONUS: 6x10 repetitions, and/or bearing on an unstable support, without support to the upper limbs.

For more information: Pr Pascal EDOUARD (Pascal.Edouard@univ-st-etienne.fr)

4. Hamstring exercises

Hamstring stretching:

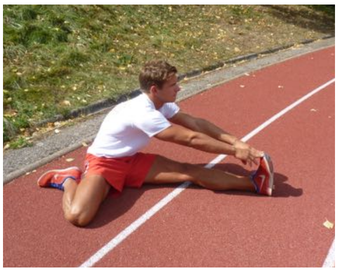	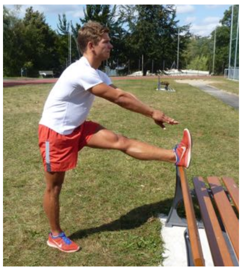	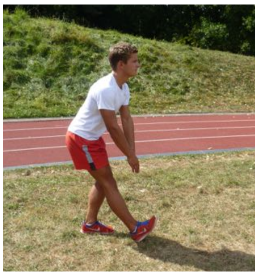
"Passing hurdles" on the ground or heel resting on a raised support, or standing with the leg stretched forward, keep an alignment between the shoulders, pelvis, knee and foot. This should not cause pain but simply a feeling of stretching. Do not try to touch your leg with your head while bending your back. Do not seek important range of motion after intense training.		
3x15 seconds on each side. Vary the positions at each session.		

Hamstring strengthening (Avoid if there is hamstring pain, aches or fatigue):

**Level 1:**	**Level 2:**	**Level 3:**	**Level 4:**	**Level 5:**
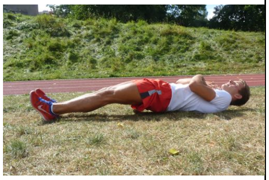	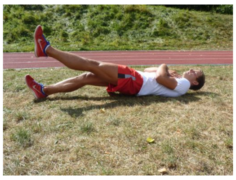	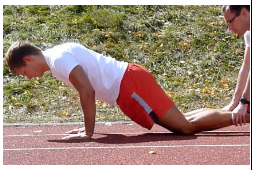	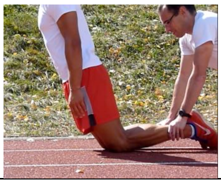	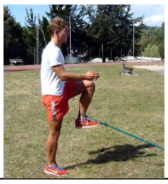
Isometric: lying on the back, lifting the buttocks and resting only on the feet and shoulders.	Isometric single leg: lying on the back, lifting the buttocks and resting only on one foot and shoulders	Eccentric: with four limbs (hands and knees) on the ground, the heels held by a fixed point, slowly release by holding by the heels, controlling the movement with the hamstrings, lightening the load by the upper limbs.	Eccentric: "Nordic hamstring": On knees, the heels held by a fixed point, the body must be completely straight from the shoulder to the knee. Lean forward as far as possible, controlling movement with the hamstrings and buttocks. Once you can not hold the position, let yourself fall by gradually putting your weight on your hands.	Pliometric: an elastic attached to a foot and a fixed point in front, perform a stride movement then return foot to initial position and retaining the advance of the leg.
6x6 seconds (R=6'')	6x6 seconds (R=6'') each side			
BONUS: 10x10 seconds (R=10'').				
Concentric / eccentric: in prone position, an elastic attached to a foot and a fixed behind, performing heel to buttocks movements (fast towards the buttocks and slow return)				
3x6 repetitions. *BONUS: 5x10 repetitions.*		1x5 repetitions, then 3x5 repetitions, then 6x6 repetitions.		

5. Achilles tendon and lower leg exercises

Lower leg stretching:

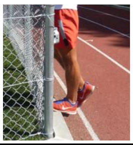 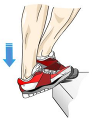	The foot on the edge of a step, the heel in the air and ankle in a dorsiflexion. This should not cause pain but simply a feeling of stretching.
	3x15 seconds each side.

Lower leg strengthening:

	**Level 1:**		**Level 2:**	
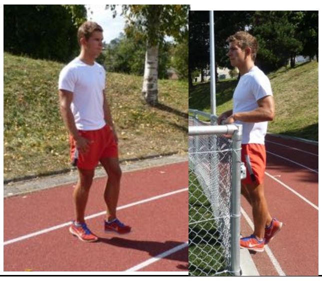	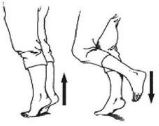	On tip toes, pass on one foot and slowly release until the heel touches the ground.	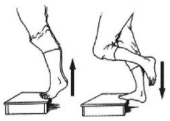	On the edge of a step or a curb, on tip toes, pass on one foot and release slowly to maximum position.
	3x8 repetitions each side.			
	BONUS: 3x10 repetitions each side, then 5x10 repetitions each side, then increase loads			
